# Hypervirulent *Klebsiella pneumoniae* Infections in Vancouver, Canada: A Clinical, Microbiological, and Genetic Retrospective Investigation

**DOI:** 10.1093/ofid/ofag654

**Published:** 2026-10-08

**Authors:** Emma C L Finlayson-Trick, Aleksandra Stefanovic, Michael Payne, Leah Gowland, Jennifer Bilawka, Gordon Ritchie, Samuel D Chorlton, Nancy Matic, Victor Leung, Laura F Mataseje, Marc G Romney, Christopher F Lowe, Patrick Tang

**Affiliations:** Department of Pathology and Laboratory Medicine, University of British Columbia, Vancouver, British Columbia, Canada; Department of Pathology and Laboratory Medicine, University of British Columbia, Vancouver, British Columbia, Canada; Division of Medical Microbiology, St. Paul's Hospital, Vancouver, British Columbia, Canada; Department of Pathology and Laboratory Medicine, University of British Columbia, Vancouver, British Columbia, Canada; Division of Medical Microbiology, St. Paul's Hospital, Vancouver, British Columbia, Canada; Division of Medical Microbiology, St. Paul's Hospital, Vancouver, British Columbia, Canada; Division of Medical Microbiology, St. Paul's Hospital, Vancouver, British Columbia, Canada; Department of Pathology and Laboratory Medicine, University of British Columbia, Vancouver, British Columbia, Canada; Division of Medical Microbiology, St. Paul's Hospital, Vancouver, British Columbia, Canada; BugSeq Bioinformatics, Inc., Vancouver, British Columbia, Canada; Department of Pathology and Laboratory Medicine, University of British Columbia, Vancouver, British Columbia, Canada; Division of Medical Microbiology, St. Paul's Hospital, Vancouver, British Columbia, Canada; Department of Pathology and Laboratory Medicine, University of British Columbia, Vancouver, British Columbia, Canada; Division of Medical Microbiology, St. Paul's Hospital, Vancouver, British Columbia, Canada; National Microbiology Laboratory, Winnipeg, Manitoba, Canada; Department of Pathology and Laboratory Medicine, University of British Columbia, Vancouver, British Columbia, Canada; Division of Medical Microbiology, St. Paul's Hospital, Vancouver, British Columbia, Canada; Department of Pathology and Laboratory Medicine, University of British Columbia, Vancouver, British Columbia, Canada; Division of Medical Microbiology, St. Paul's Hospital, Vancouver, British Columbia, Canada; Department of Pathology and Laboratory Medicine, University of British Columbia, Vancouver, British Columbia, Canada; Division of Medical Microbiology, St. Paul's Hospital, Vancouver, British Columbia, Canada

**Keywords:** antimicrobial resistance, Hypervirulent *Klebsiella pneumoniae*, string test, whole genome sequencing

## Abstract

**Background:**

Hypervirulent *Klebsiella pneumoniae* (hvKp) is poorly characterized in North America and likely underrecognized. We evaluated a molecular screening approach for hvKp and described the clinical, microbiological, and genomic characteristics of hvKp isolates in Vancouver, Canada.

**Method:**

We conducted a retrospective study of sterile-site Kp isolates recovered at a tertiary care center (2018–2024). Archived isolates were screened using polymerase chain reaction (PCR) targeting *peg-344* and *iucA*. Whole-genome sequencing was then performed, with hvKp defined as a Kleborate virulence score of ≥3. Clinical data were obtained through chart review. Genomic relatedness was assessed using reference-based multilocus sequence typing.

**Results:**

Among 449 isolates, 43 were PCR positive for *peg-344* and/or *iucA*, and 38 met criteria for hvKp. Only 5 (13%) cases were suspected at the time of isolation. The molecular screen demonstrated 100% sensitivity and 98.8% specificity for hvKp detection. Compared with classical Kp, hvKp infection was independently associated with travel history (adjusted odds ratio [aOR], 9.73; 95% CI, 2.06–46.04) and residence in Vancouver's Downtown Eastside (DTES; aOR, 3.69; 95% CI, 1.05–12.97), but not with Indo-Pacific country of origin (aOR, 1.77; 95% CI, .49–6.37). Genomic analysis suggested possible local transmission between 2 DTES patients. K1/K2 were the most common capsular serotypes. One ST15 hvKp isolate carried *bla*_NDM_, representing a convergent hypervirulent and carbapenem-resistant strain.

**Conclusions:**

Routine molecular screening improved detection of hvKp and revealed evolving epidemiology, including possible clonal transmission. Implementing laboratory-based molecular screening can enhance clinical recognition, guide evaluation for metastatic complications, and monitor the emergence of high-risk strains.

## Teaser

Hypervirulent *Klebsiella pneumoniae* is likely underrecognized and poorly characterized in Canada. This study assessed a molecular screening method and detailed the clinical, microbiological, and genomic features of isolates identified in a tertiary care hospital in Vancouver, British Columbia.

Infections due to *Klebsiella pneumoniae* (Kp) cause significant morbidity and mortality worldwide [1]. In Canada, Kp is one of the most frequently isolated bacterial pathogens in people who are hospitalized patients [2]. *Klebsiella pneumoniae* has 2 distinct pathotypes: classical (cKp), which primarily causes healthcare-associated infections, and hypervirulent (hvKp), which can affect otherwise healthy individuals and cause community-onset liver abscess with potential metastatic spread to sites, such as the eyes, central nervous system, and lungs [3, 4]. Given the severity of hvKp infections, timely and accurate diagnosis is essential.

Although first described in a case series from Taiwan, hvKp has now disseminated worldwide [5–8]. North American data on the prevalence and clinical characteristics of hvKp remain limited. In a population-based surveillance study from Calgary (2001–2007), 8.2% of bloodstream Kp isolates exhibited a hypermucoviscous phenotype, with the majority testing positive for *rmpA*; however, this phenotype alone does not reliably distinguish hvKp from classical strains [9]. In the United States, publications from New York and Chicago, which used more comprehensive definitions of hvKp, observed prevalence rates of 3.5% and 3.8%, respectively [10, 11]. Otherwise, clinical descriptions of hvKp have been primarily published as case reports [12, 13]. As such, contemporary data describing hvKp in North America is needed.

There is no definitive diagnostic test for hvKp. Diagnosis has historically relied on a combination of clinical findings, laboratory demonstration of hypermucoviscosity (ie, string test), and detection of virulence-associated genes [3]. This approach has some limitations as not all hvKp isolates demonstrate hypermucoviscosity and there is no consensus on which virulence genes to evaluate in routine testing [14–16]. In Canada, clinical laboratories refer suspected hvKp isolates to the reference National Microbiology Laboratory (NML) for polymerase chain reaction (PCR) -based detection of virulence genes; however, referral depends on clinical suspicion, raising the possibility of underrecognition. These challenges highlight the need for hospital-based molecular screening to enable timely identification of hvKp and to guide appropriate clinical investigation and management.

In this retrospective study, we aimed (1) to evaluate a PCR screen targeting *peg-344* and *iucA* for identifying hvKp from sterile-site isolates collected between 2018 and 2024 at a tertiary care center and (2) to characterize the clinical, microbiological, and genomic features of these hvKp isolates.

## METHODS

### Historical Practice

Any Kp isolate that demonstrated a hypermucoviscous phenotype via a string test in the clinical microbiology laboratory, or that was obtained from a clinical presentation suspicious for hvKp, was referred to the NML for further characterization. At the NML, isolates underwent PCR testing for K1-, K2-, and K5-serotype-specific genes, as well as selected virulence genes, including *rmpA*, *rmpA2*, *iucA*, *iroB*, *ybtA*, and *clbA* [17]. The NML does not apply a formal definition of hypervirulence; rather, results are reported as the presence or absence of individual serotype and virulence genes.

### Screening and Sequencing Archived *Klebsiella pneumoniae* Isolates

All frozen Kp isolates recovered from sterile site specimens by the St. Paul's Hospital Microbiology Laboratory between 1 October 2018 and 2024 were screened via PCR targeting *peg-344* and *iucA* (Table 1) [18].

**Table 1.** Primers and Probes Used for Detection of *peg-344* and *iucA*

Name	Sequence (5′ → 3′)
peg-344 forward	AGCTTCACTTTCGTTCATCCT
peg-344 reverse	CTGCAGAAGAAAGGGCAATAAC
peg-344 probe	FAM-TCCACTGGCTTTCTGTCCTTTCCC-BHQ1
iucA forward	TTGTCGCTAAAGGGCTGATT
iucA reverse	CACGCTCAGGGAGAATTTGA
iucA probe	VIC-ACCTCTTCCCGCTCGCTCTACT-BHQ1

### Whole-Genome Sequencing and Bioinformatic Analysis

All isolates that screened positive for *peg-344* and/or *iucA* (*N* = 43) underwent whole-genome sequencing (WGS). Frozen isolates were cultured overnight in Mueller–Hinton broth. Bacterial DNA was then extracted using the MagNA Pure 24 (Roche Diagnostics, Basel, Switzerland). A sequencing library was prepared using the SQK-NBD114.24 kit (Oxford Nanopore Technologies, Oxford, UK). Sequencing was performed on the GridION Mk1 (Oxford Nanopore Technologies) using R10.4.1 flow cells (depth of coverage, range 32–111×; Q score, range 18–22). Reads were filtered before analysis for length longer than 40 base pairs and average quality greater than Q7. Data acquisition and base calling into FASTQ files were completed using Guppy version 6.3.9 (Oxford Nanopore Technologies). FASTQ files were analyzed by BugSeq.com (Vancouver, Canada). For plasmid analysis, BugSeq uses MOB-suite with a curated database to remove erroneous sequences and improve accuracy [19]. For antimicrobial resistance (AMR) analysis, BugSeq uses a proprietary database of over 8000 AMR genes, mutations, insertion sequences, and knockouts.

Isolates were assigned a virulence score (0–5) based on the Kleborate virulence scoring system [20]. In short, the virulence scoring system is based on the presence or absence of yersinabactin (*ybt*), colibactin (*clb*), and aerobactin (*iuc*). A score of 3 corresponds to the presence of *iuc* alone, a score of 4 to *iuc* plus *ybt*, and a score of 5 to the presence of all 3 genes. Isolates with a virulence score of ≥ 3 were classified as hvKp, whereas those with scores of 0–2 were classified as cKp [21]. Isolates classified as cKp were randomly selected for inclusion as controls to achieve the desired sample size. To determine the required number of controls for our fixed cohort of 38 hvKp isolates, a power calculation was performed for a comparison of 2 independent proportions. Assuming a 2-sided alpha of 0.05 and 80% power, an allocation ratio of approximately 3.4:1 (129 cKp–38 hvKp) was required to detect a minimum absolute difference of 19.5% for rare clinical features (baseline prevalence of 5%) and up to 29.5% for more common baseline characteristics (prevalence of 30%). Based on this calculation, 129 cKp patient charts were randomly selected for inclusion and review.

Reference-based multilocus sequence typing (refMLST) was performed on all hvKp isolates using the National Center for Biotechnology Information (NCBI)RefSeq assembly GCF_000240185.1 Kp genome as the reference sequence. The threshold of <20 allele differences by refMLST was previously validated to indicate epidemiological relatedness or clonality and corresponds to <10 alleles by core genome MLST [22].

### Chart Review

For both people with hvKp and cKp, clinical data were extracted from electronic medical records and included patient age, sex, diabetes history, travel history (outside of British Columbia in the 5 years before presentation to hospital), Indo-Pacific country of origin (China, Japan, North Korea, South Korea, Taiwan, Philippines, Vietnam, Cambodia, Thailand, Malaysia, Singapore, Brunei, and Indonesia), and postal code. For people with hvKp, additional information was collected, including presenting symptoms, treatment with antibiotics (type and duration), length of admission (days), intensive care unit (ICU) admission, sites of dissemination, liver abscess, time to specialist consultation, mortality, and microbiological investigations.

### String Test

All hvKp isolates were evaluated using the string test. In brief, frozen isolates were thawed and subbed twice on 5% sheep blood agar. After 24-hour incubation at 37°C, an inoculating loop was used to gently stretch individual colonies. Isolates were considered string test positive if the colony stretched >5 mm.

### Antimicrobial Susceptibility Testing

Antimicrobial susceptibility testing was performed using the VITEK 2 system (bioMérieux, Marcy-l’Étoile, France) and interpreted according to Clinical and Laboratory Standards Institute M100 standards ED35:2025 [23]. Antibiotics tested included cefazolin, ceftriaxone, ciprofloxacin, imipenem, meropenem, ertapenem, piperacillin–tazobactam, trimethoprim–sulfamethoxazole, and tobramycin.

### Statistical Methods

Statistical analyses were performed using Microsoft Excel and jamovi software (version 2.6) [24]. For univariate analysis, pairwise deletion was used, restricting exclusions only to the specific variables with missing data. Categorical variables were compared using the χ² test or Fisher's exact test, as appropriate, while continuous variables were compared using Student's *t*-test. A *P* value of <.05 was considered statistically significant. For the multivariate analysis, a binomial logistic regression model was constructed using variables selected a priori based on clinical and epidemiological relevance (age, sex, diabetes, travel history, Indo-Pacific origin, and DTES residence). Isolates with missing data were excluded. To isolate baseline risk factors and avoid confounding, variables consequent to infection (liver abscess and ICU admission) were excluded, and all preinfection characteristics were entered simultaneously regardless of univariate significance.

### Patient Consent Statement

Ethics approval was obtained, and informed consent was waived by the University of British Columbia/Providence Health Care Research Ethics Board (H24-03595).

## RESULTS

### Screening

During the study period, 449 Kp isolates were recovered from sterile sites. Of these, 43 were positive for *peg-344* and/or *iucA* by PCR, and 38 (88.3%) had a Kleborate score of ≥3, meeting criteria for hvKp. Five of the 43 screen-positive isolates had a Kleborate score of <3, and these were excluded from further analysis. At the time of initial isolation, only 5 cases were clinically or microbiologically suspected as hvKp and referred to the NML; all 5 were subsequently confirmed as hvKp. The 406 screen-negative isolates did not undergo WGS based on the rationale that, lacking *iucA*, their maximum Kleborate score would be 2. Using a Kleborate score of ≥3 as the reference standard, the screen had a sensitivity of 100%, a specificity of 98.8%, a positive predictive value of 88.4%, and a negative predictive value of 100%.

### Clinical Characteristics

Among 167 people, 38 were classified as hvKp and 129 as cKp based on Kleborate scoring. In Table 2, people with hvKp were more likely to be of Indo-Pacific origin (53% vs 30%, *P* = .017) and to have a recent travel history (29% vs 7.8%, *P* = .003). There were no significant differences in age, sex, diabetes history, all-cause in-hospital mortality, or residence in Vancouver's Downtown Eastside (DTES). In multivariate analysis (Table 3), residence in the DTES (adjusted odds ratio [aOR], 3.69; 95% CI, 1.05–12.97; *P* = .04) and travel history (aOR, 9.73; 95% CI, 2.06–46.04; *P* = .004) were independently associated with hvKp. Other variables, including age, sex, diabetes, and Indo-Pacific origin, were not significantly different between hvKp and cKp in multivariate analysis.

**Table 2.** Univariate Analysis Comparing Demographic and Clinical Characteristics Between People With hvKp and cKp

	HvKp, *N* = 38	CKp, *N* = 129	*P* Value
Age, median (IQR)	66 (58–75)	69 (61–82)	.28
Male sex, *n/N* (%)	29/38 (76)	89/129 (69)	.38
Diabetes, *n/N* (%)	11/38 (29)	39/129 (30)	.88
DTES postal code, *n/N* (%)	9/38 (24)	17/129 (13)	.16
Indo-Pacific country of origin, *n/N* (%)	17/32 (53)	28/94 (30)	.017
Travel history, *n/N* (%)	7/24 (29)	9/116 (7.8)	.003
All-cause in-hospital mortality, *n/N* (%)	7/38 (18)	28/129 (22)	.66

Pairwise deletion was used, restricting exclusions only to the specific variables with missing data. *P* values of <.05 were considered statistically significant. DTES refers to Downtown Eastside (postal code V6A). Data are presented as median (interquartile range, IQR) for age and *n* (%) for categorical variables.

**Table 3.** Multivariate Analysis Comparing Demographic and Clinical Characteristics Between People With hvKp (*N* = 20) and cKp (*N* = 87)

Variable	Adjusted OR (95% CI)	*P* Value
Age	0.99 (.96–1.04)	.97
Male sex	2.84 (.61–13.27)	.18
Diabetes	0.79 (.22–2.77)	.71
DTES postal code	3.69 (1.05–12.97)	.04
Indo-Pacific country of origin	1.77 (.49–6.37)	.38
Travel history	9.73 (2.06–46.04)	.004

Isolates with missing data were excluded from the binomial logistic regression testing. Adjusted odds ratios (aORs) with 95% CIs are shown. *P* values of <.05 were considered statistically significant. DTES refers to Downtown Eastside (postal code V6A).

For people with hvKp (*N* = 38), most presented from the community (*N* = 32, 84%). The main presenting complaints included fever (*N* = 23, 61%), weakness and fatigue (*N* = 17, 45%), and gastrointestinal symptoms (*N* = 16, 42%). Almost all people were bacteremic (*N* = 36, 95%). People had other sterile cultures that were positive for Kp, including peritoneal fluid (*N* = 1), prostate abscess (*N* = 1), and renal abscess (*N* = 1). Notably, 1 person had bacteremia with Kp and vision loss in the right eye. A vitreous fluid specimen from that person was culture negative but ultimately tested positive for Kp via 16S rRNA sequencing. There were no other people with endophthalmitis. Eight people had liver involvement with an average abscess volume of 239 cc, and 7 underwent drainage. The hospital Infectious Diseases service was consulted in 18 cases (47%). The most commonly used inpatient antibiotic was piperacillin–tazobactam (*N* = 28, 74%). The median length of stay in the hospital was 13 days (range, 1–190 days). Twelve people (31.6%) required ICU admission. Seven people died while admitted (18%), 2 within the first 24 hours, and 2 people died in the 30 days following discharge. In comparison, 36 people with cKp (28%) died during their hospital stay (*N* = 28) or within the 30 days following discharge (*N* = 8).

### Microbiology

As hypermucoviscosity is commonly associated with hvKp and has been linked to regulators of mucoid phenotype *rmpA* and *rmpA2*, the association between these genetic markers and string test positivity was evaluated. The string test was completed on 36 hvKp isolates (2 isolates failed to grow), of which 28 (78%) were string test positive. Through genomic analysis, 23 of the 28 string test-positive isolates were positive for a functional copy of *rmpA* only, and 5 did not have functional copies of either gene. Of the 8 string test-negative isolates, 2 were positive by genomic analysis for *rmpA* alone, and 6 lacked functional copies of either genes. Using the presence of *rmpA/rmpA2* as the reference gold standard, the string test demonstrated a sensitivity of 92%, a specificity of 54.5%, a positive predictive value of 82.1%, and a negative predictive value of 75%.

The majority of isolates (*N* = 27, 63%) were susceptible to all routinely tested antibiotics. Only 1 isolate demonstrated resistance to all antibiotics tested, both phenotypically and genotypically, carrying *bla*_CTX-M-15_ and *bla*_NDM_. Among the remaining isolates, 2 that were phenotypically resistant to cefazolin harbored *bla*_SHV-33_ and *bla*_SHV_ (C112A), and 3 that were resistant to trimethoprim–sulfamethoxazole carried *dfrA50/14* and *sul2*.

### Genomic

Genomic analysis (*N* = 38) identified a diverse array of plasmids, predominantly belonging to the IncFIB and IncFII replicon types and AMR genes; details for all isolates are provided in Supplementary Table 1. The capsular serotype (K) and sequence type (ST) of hvKp strains are shown in Figure 1. Seven serotypes were present in the dataset, with K2 (*N* = 14, 37%), K1 (*N* = 12, 32%), and K20 (*N* = 6, 16%) accounting for the majority of isolates. All K1 serotype isolates belonged to ST23, whereas K2 and K20 contained a variety of ST types, including ST65 (*N* = *3,* 7%), ST66 (*N* = *3,* 7%), ST86 (*N* = *3,* 7%), and ST268 (*N* = *3,* 7%). The isolate carrying *bla*_NDM_ was part of ST15-K2 and was string test negative.

**Figure 1.**
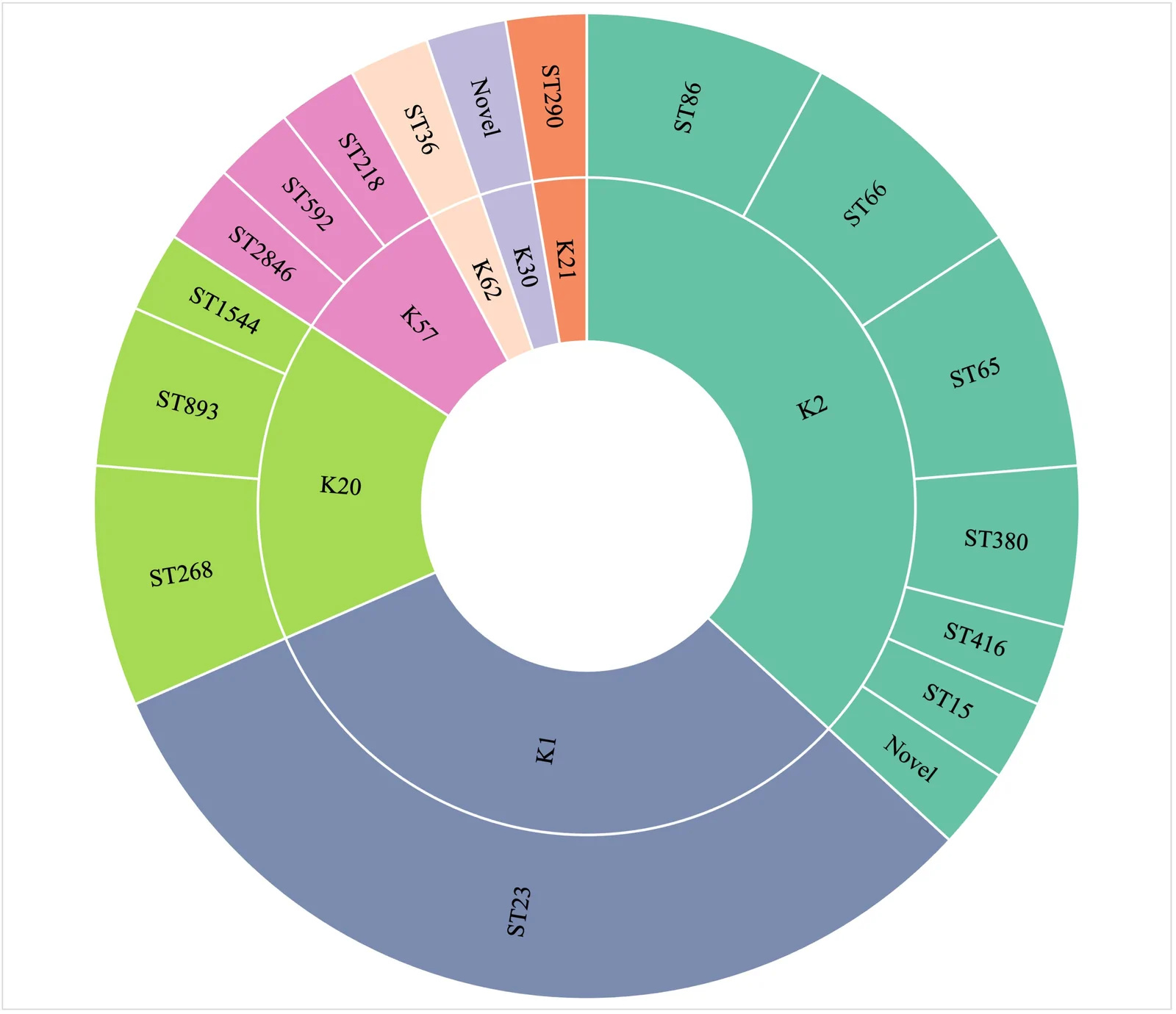
The capsular serotype (K) and sequence type (ST) of hvKP strains. K1 (predominantly ST23) and K2 (including ST65, ST66, ST86, ST380, ST416, and ST15) are the most common. Other capsular types include K20, K57, K62, K30, and K21 with their respective sequence types. Segment size reflects the relative number of isolates.

Genetic relatedness of hvKp isolates was assessed using refMLST. Two isolates differed by fewer than 20 alleles, suggesting clonality. Both isolates were ST66-K2. Epidemiologic review revealed that these people resided in similar areas of Vancouver's DTES, indicating possible local transmission. Otherwise, the refMLST analysis was genetically diverse.

### Description of Excluded Cases

Five screen-positive isolates were excluded from downstream analyses due to a Kleborate virulence score of <3. Clinically, however, these cases demonstrated features commonly associated with hvKp infection. Four of five people were male (age range, 53–79 years), and 3 had diabetes. Two people were originally from Indo-Pacific countries with recent travel to those regions. Four people required hospital admission, including 1 ICU admission. Two people developed liver abscesses, one of whom underwent drainage. All 5 isolates were string test positive. In addition, most isolates (*N* = 4) were negative for other virulence genes, including *clbA* and *ybtA*.

## DISCUSSION

Here, we describe the evolving clinical and molecular epidemiology of hvKp in a Canadian tertiary care setting while identifying important gaps in current diagnostic practice. Duplex PCR targeting *iucA* and *peg-344* markedly improved detection, identifying 33 additional hvKp cases missed by conventional screening. Of note, these missed cases tended to be before 2023, when there may have been less clinical awareness of hvKp in our region. These findings suggest that hvKp is underrecognized in Vancouver.

Historically, clinical laboratories have relied on the string test as a surrogate marker of hypervirulence. Our findings highlight the limitation of this approach. Although the string test demonstrated moderate sensitivity (92%), the low specificity (54.5%) and low negative predictive value (75%) render it unreliable as a primary screening tool for hvKp. Consistent with Wahl et al, we observed discordance between the hypermucoviscous phenotype and the presence of *rmpA* and/or *rmpA2*, with 2 of 8 string test-negative isolates containing *rmpA.* Kochan et al described this result further, demonstrating how mutations in *rmpA* and/or *rmpA2* either impaired gene expression or function, leading to isolates that were not hypermucoviscous [25]. Conversely, the 5 isolates excluded due to Kleborate virulence scores of <3 were all string test positive and harbored *rmpA*. However, these isolates lacked several additional virulence genes, suggesting that they may represent a hypermucoviscous phenotype without hypervirulence. Given the clinical presentation of these hypermucoviscous strains with lower virulence scores, they may still warrant additional clinical attention. Collectively, these results reinforce that hypermucoviscosity and hypervirulence represent overlapping, but distinct, phenotypes and that reliance on phenotypic traits alone can lead to underdetection of hvKp [14].

In contrast, genotypic screening targeting *peg-344* and *iucA* is sensitive (100%) and specific (98.8%). Although the precise function of the metabolic transporter encoded by *peg-344* remains incompletely characterized, its presence, particularly in conjunction with the aerobactin siderophore biosynthesis gene *iucA*, is strongly predictive of the hvKp pathotype [26]. All of our hvKp isolates were positive for *iucA* by PCR. Our duplex PCR assay independently validates the findings of Duan et al (2024) within a Canadian clinical cohort, supporting a transition toward molecular-based screening for hvKp.

Beyond diagnostics, our data reveal a shifting epidemiological profile of hvKp in Vancouver. While travel remained a robust predictor, residence within the DTES emerged as a strong, independent risk factor for hvKp in both the univariate and multivariate analyses. Characterizing the specific demographics of the DTES remains challenging as transient or marginalized populations are frequently underrepresented in traditional surveillance. However, data from the 2021 Canadian Census for the V6A postal code provides some context: While approximately 29% of respondents identified as Indo-Pacific, a population historically linked to clonal hvKp lineages, the vast majority (61.4%) were nonimmigrants [27]. This dual profile, combined with our observation of 2 people infected with a clonal strain, suggests transmission independent of recent travel. The high-density living environments characteristic of the DTES, including shelters and single-room occupancy hotels, may facilitate hvKp transmission in a manner analogous to previously documented intrafamilial clusters [28, 29]. These findings suggest that hvKp is no longer exclusively an infection linked to international travel or country of residence but could be circulating within local urban networks.

Overall, AMR among the isolates was limited; however, the emergence of carbapenem resistance in hvKp is of particular concern. There are 3 main evolutionary pathways for this to occur: hvKp strains acquire carbapenem resistance plasmids (ie, carbapenemase-producing ancestral hvKp; eg, ST23), carbapenem-resistant Kp strains acquire virulence plasmids (ie, convergent hypervirulent carbapenem-resistant Kp; eg, ST15), or a single plasmid acquires both carbapenem resistance and virulence and is carried in either strain [30]. In our data, the 12 ST23 isolates did not demonstrate significant phenotypic or genotypic resistance. Nevertheless, ST23 has a strong capacity to acquire carbapenem resistance, which is in part why the World Health Organization assessed the global risk of hvKp as moderate [31]. We did, however, identify a single isolate harboring both hypervirulence determinants and carbapenem resistance, belonging to ST15, a globally disseminated multidrug-resistant lineage [32–34]. This convergence of hypervirulence and carbapenem resistance parallels clinically significant reports from other regions, including a recent study from Alberta, Canada describing carbapenem resistance in 16 hvKp isolates [17]. Strains combining hypervirulence with carbapenem resistance represent a critical public health threat because they are capable of causing severe, invasive disease in otherwise healthy individuals while evading first-line therapies.

These findings have direct implications for routine clinical microbiology practice at the institutional level. In our setting, hvKp testing has historically been triggered by clinician suspicion or phenotypic features, such as hypermucoviscosity, a strategy that failed to identify a substantial proportion of cases in our study. Implementation of routine molecular screening for hvKp among Kp isolates from normally sterile sites would provide earlier and more reliable recognition of hypervirulent strains. Such early identification could serve as an actionable laboratory alert, prompting heightened clinical vigilance and targeted evaluation for metastatic complications, including endophthalmitis and central nervous system infection. While the identification of hvKp does not inherently alter empirical treatment duration for uncomplicated bacteremia, the discovery of metastatic foci dictates the necessity for prolonged therapy and timely source control, including drainage of hypermucoviscous collections [35].

This study has a few notable limitations. First, as there is no consensus definition for hvKp, we defined hvKp using a Kleborate virulence score of ≥3, corresponding to the presence of aerobactin with or without colibactin and yersiniabactin. We acknowledge that some studies define hvKp by the presence of 5 virulence-associated genes (*peg-344*, *rmpA*, *rmpA2*, *iroB*, and *iucA*) [36, 37]. However, applying this definition to our dataset would have reclassified 12 isolates as cKp. Given our objective of maximizing case ascertainment for epidemiologic and clinical characterization, a sensitive screening strategy was intentionally prioritized. Second, the retrospective, single-center design may limit generalizability to other settings. To mitigate selection bias in the chart review, we included people with cKp from the same hospital; however, residual confounding related to healthcare utilization and social determinants of health may have persisted. Third, screening was limited to body fluids from sterile sites, and exclusion of nonsterile samples may have resulted in underestimation of hvKp prevalence, although prior studies suggest that most people present with bacteremia or abscess [38]. Finally, we did not test virulence using a mouse model as this modality was not available for our clinical laboratory [25, 36].

## CONCLUSION

In this Canadian tertiary care setting, molecular screening using *iucA* and *peg-344* substantially improved detection of hvKp compared with phenotypic features. Our findings also indicate a changing clinical epidemiology, with hvKp no longer confined to traditional risk groups and evidence suggestive of local transmission within Vancouver's DTES. Routine molecular detection may therefore enhance both clinical recognition and understanding of hvKp circulation in urban settings.

## Supplementary Material

ofag654_Supplementary_Data

## Supplementary Data

Supplementary materials are available at *Open Forum Infectious Diseases* online. Consisting of data provided by the authors to benefit the reader, the posted materials are not copyedited and are the sole responsibility of the authors, so questions or comments should be addressed to the corresponding author.
