# Supplementary material for: Hypervirulent Klebsiella pneumoniae Infections in Vancouver, Canada: A Clinical, Microbiological, and Genetic Retrospective Investigation
Source: Open Forum Infect Dis. 2026 Oct 8;13(10):ofag654. doi: 10.1093/ofid/ofag654 (PMC13660691; doi:10.1093/ofid/ofag654)
Supplement: Supplementary file 1 [file ofag654_supplementary_data.docx]

**Supplementary Table 1.** Isolate, Kleborate score, capsular serotype, sequence type, plasmid replicons, and chromosomal/intrinsic antimicrobial resistance (AMR) genes/mutations for hvKp isolates (*N=*38) from a tertiary care hospital in Vancouver, Canada between 1 October 2018 and 2024.

| **Isolate** | **Kleborate score** | **Capsular serotype** | **Sequence type** | **Plasmid replicon(s)** | **AMR genes/mutations** |
| --- | --- | --- | --- | --- | --- |
| hvKP01 | 5 | K20 | 268 | IncFIB; IncFII; IncHI1B | none |
| hvKP04 | 5 | K1 | 23 | IncFIA; IncFII | none |
| hvKP05 | 5 | K1 | 23 | IncFIB; IncHI1B | none |
| hvKP06 | 3 | K1 | 23 | IncFIB; IncFII; IncHI1B | none |
| hvKP07 | 3 | K2 | 380 | IncFIA; IncFIB; IncFII | *oqxA*, *oqxB*, *ompK36* (p.L312*), *bla*_SHV-33_ |
| hvKP08 | 5 | K2 | 66 | IncFIA; IncFIB; IncFII | none |
| hvKP09 | 5 | K1 | 23 | IncFIA; IncFIB; IncFIC;IncFII; IncHI1B | *dfrA50* |
| hvKP10 | 5 | K1 | 23 | ColRNAI | none |
| hvKP11 | 5 | K2 | 65 | IncFIB; IncFII; IncHI1B | none |
| hvKP12 | 5 | K57 | 2846 | ColRNAI, IncFIB; IncFII | none |
| hvKP13 | 5 | K1 | 23 | IncFIB; IncHI1B | none |
| hvKP14 | 5 | K1 | 23 | ColRNAI; IncFIB; IncHI1B | none |
| hvKP15 | 5 | K2 | 65 | IncFIB; IncFII; IncHI1B | none |
| hvKP16 | 4 | K2 | 380 | IncFIB; IncFII | none |
| hvKP17 | 5 | K1 | 23 | ColRNAI; IncFIB; IncHI1B | none |
| hvKP18 | 5 | K2 | 65 | IncFIB; IncFII; IncHI1B | none |
| hvKP19 | 5 | K20 | 268 | IncFIB; IncFII; IncHI1B | *bla*_SHV_ (C-112A) |
| hvKP20 | 5 | K1 | 23 | IncFIB; IncFII; IncHI1B; IncN; IncR | *qnrS1*, *aph(3’)-Ib*, *dfrA14*, *sul2* |
| hvKP21 | 5 | K20 | 268 | IncFIB; IncFII; IncHI1B | none |
| hvKP22 | 5 | K1 | 23 | IncFIA; IncFIB; IncFIC; IncHI1B | none |
| hvKP23 | 5 | K2 | 66 | IncFIA; IncFIB; IncFII | none |
| hvKP24 | 5 | K2 | 66 | IncFIA; IncFIB; IncFII | none |
| hvKP25 | 5 | K1 | 23 | ColRNAI; IncFIB; IncHI1B | *mpmE* |
| hvKP26 | 5 | K62 | 36 | ColRNAI; IncFIB; IncFII; IncHI1B | none |
| hvKP27 | 5 | K21 | 290 | none | *bla*_LAP-2_, *qnrS1*, *sul2*, *tet(A)* |
| hvKP28 | 3 | K20 | 1544 | IncFIB; IncFII; IncHI1B | *dfrA50* |
| hvKP29 | 3 | K2 | 416 | ColRNAI; Col(MG828); IncFIB; IncFII; IncI-gamma/K1 | *bla*_LAP-2_, *aph(3’)-Ib*, *aph(6’)-Id*, *floR*, *qnrS1*, *tet(A)*, *sul2* |
| hvKP30 | 3 | K30 | novel | ColRNAI; IncFIB; IncFII | *bla*_TEM-1_ |
| hvKP31 | 3 | K1 | 23 | IncFIB; IncFII; IncHI1B | *qnrS1*, *tet(A)*, *dfrA1*, *sul1* |
| hvKP32 | 3 | K2 | 86 | IncFIB; IncFII; IncHI1B | none |
| hvKP33 | 3 | K20 | 893 | IncFIB; IncFII; IncHI1B | *dfrA50* |
| hvKP34 | 3 | K20 | 893 | IncFIB; IncFII; IncR | *mcr-8.2*, *dfrA50* |
| hvKP35 | 3 | K2 | 15 | ColRNAI; IncC; IncFIB; IncFII; IncHI1B | *bla*_CMY-6_, *bla*_NDM-1_, *bla*_CTX-M-15_, *armA*, *rmtC, aac(6')-Ib3*, *aog(3')-Ia*, *dfrA1*, *dfrA12*, *sul1*, *gyrA* D87A, *gyrA* S83F, *parC* S80I, *tet(A)*, *ramR* A19V |
| hvKP36 | 3 | K2 | 86 | IncFIB; IncFII; IncHI1B | none |
| hvKP37 | 3 | K2 | novel | IncFIB; IncFII; IncHI1B | none |
| hvKP38 | 3 | K57 | 592 | Col(Ye4449); IncFIB; IncHI1B | none |
| hvKP39 | 3 | K57 | 218 | IncFIB; IncFII; IncHI1B | none |
| hvKP43 | 3 | K2 | 86 | ColRNAI; IncFIB; IncFII; IncHI1B | *ΔcirA* |
